# Cellular and molecular immunomodulatory potential of red wine polyphenols in apical periodontitis

**DOI:** 10.1590/1414-431X2025e15099

**Published:** 2026-01-30

**Authors:** R. Ricci, B.M. Pereira, J.D.A. Alvarado, R.O. Sales-Junior, N.E.S. Machado, D.C. dos Santos, F.H.M. Pederro, M.C. Ferraz, S.C.T. Frasnelli, J.S.M. Rodrigues, S.H.P. Oliveira, L.T.A. Cintra, A. Kishen, J.E. Gomes-Filho

**Affiliations:** 1 Departamento de Odontologia Preventiva e Restauradora, Faculdade de Odontologia, Universidade Estadual Paulista, Araçatuba, SP, Brasil; 2 Departamento de Clínica, Cirurgia e Reprodução Animal, Faculdade de Medicina Veterinária, Universidade Estadual Paulista, Araçatuba, SP, Brasil; 3 Departamento de Ciências Básicas, Faculdade de Odontologia, Universidade Estadual Paulista, Araçatuba, SP, Brasil; 4 Department of Dentistry, Faculty of Dentistry, University of Toronto, Toronto, ON, Canada

**Keywords:** Apical periodontitis, Bone remodeling, Dealcoholized red wine, Inflammation, Red wine

## Abstract

This *in vitro* and *in vivo* study assessed dealcoholized red wine (DRW) effects on cytokine profile in macrophage (MQ)-periodontal ligament fibroblast (PDLFs) co-cultures and its impact on blood parameters, inflammatory/bone markers, cytokine expression, and periapical bone loss in rat apical periodontitis (AP). A MQ-PDLFs co-culture and Wistar rats with induced AP were exposed to DRW or red wine (W), with DMEM or water as controls (C). Cell cultures were analyzed for cytokine profile using a multiplex immunoassay. Rats underwent blood profiling, radiography, qRT-PCR, and histometric analysis of AP. Statistical significance was set at 5%. Multiplex analysis of the co-culture revealed that DRW induced lower interleukin (IL)-6 and tumor necrosis factor (TNF)-α levels compared to C and W, higher IL-10 level than C, and lower IL-1β level only compared to W (P<0.05). Radiographic images confirmed AP development in rats. DRW showed a reduced monocyte count compared to C (P<0.05), but the inflammatory/bone markers in plasma were similar (P>0.05). Additionally, DRW showed lower IL-1β expression than C, and higher IL-10 expression only compared to W in AP (P<0.05). Periapical bone loss was similar among groups (P>0.05). In conclusion, DRW promoted an anti-inflammatory profile in co-cultures and *in vivo*. However, these effects did not translate into differences in lesion size or bone loss within the experimental model evaluated.

## Introduction

Apical periodontitis (AP) is an inflammatory condition affecting the periapical tissues of teeth with necrotic pulps, primarily triggered by bacterial infection and their by-products (1). Pulpal necrosis allows microbial invasion to extend into the periodontal ligament and alveolar bone, initiating an immune response. This response involves both innate and adaptive mechanisms, characterized by the recruitment of inflammatory cells to the lesion site, where they release key cytokines that drive inflammation and osteoclastic activity, ultimately leading to bone resorption (2,3).

The progression of AP reflects a disruption in the balance between inflammation and tissue repair, resulting in sustained periapical damage and continued immune activation (4). Multiple foci of AP have been associated to significant systemic repercussions, including altered blood parameters and disrupted immune homeostasis, with elevated plasma levels of pro-inflammatory cytokines and reduced levels of anti-inflammatory cytokines (5-9).

A diet rich in polyphenols has been linked to anti-inflammatory and antioxidant responses in various pathological conditions (10,11). Although red wine (W) is widely studied for these benefits, its health-promoting effects are primarily attributed to its polyphenolic rather than the alcoholic content (12-14). In this context, dealcoholized red wine (DRW) emerges as a promising alternative, offering the polyphenols without the health risks and inflammation associated with alcohol consumption (15-17).

Studies have demonstrated that DRW can exert anti-inflammatory effects in various inflammatory conditions (18,19). Previous findings revealed that supplementation with DRW reduced inflammation and bone loss in rats with induced AP (13,14). All together, these results suggest that DRW may modulate the inflammatory response in AP through mechanisms associated with polyphenol activity. However, the cellular and molecular pathways underlying DRW effects remain to be elucidated.

Therefore, the present study aimed to clarify some of these pathways by investigating the immunomodulatory potential of DRW and W in the context of AP. The cytokine and chemokine profile of macrophages (MQ) and periodontal ligament fibroblasts (PDLFs) in direct co-culture with DRW or W exposure was first examined *in vitro*, allowing juxtacrine interactions. In parallel, an *in vivo* model was used to evaluate both systemic and local effects of DRW or W supplementation, including changes in blood profile, inflammatory and bone metabolism markers in plasma, cytokine expression at the lesion site, and histometric analysis of periapical bone loss. We hypothesized that DRW and W differentially influence the inflammatory profile in AP by modulating cytokine production and immune cell recruitment both locally and systemically.

## Material and Methods

### Cell cultures

THP-1 monocytes (ATCC TIB-202; American Type Culture Collection, USA) were cultured in complete RPMI 1640 medium (#A104950; Gibco, USA), supplemented with 10% heat-inactivated fetal bovine serum (#19C448; Sigma-Aldrich, USA), 1% antibiotic/antimycotic (#15240-062, Gibco), and 0.1% beta-mercaptoethanol (#21985023, Gibco). The cells were incubated at 37°C in a humidified atmosphere containing 5% CO_2_. Following this, THP-1 cells (2.5×10^5^ cells/mL) were differentiated into MQ by exposure to 100 nmol/L phorbol 12-myristate-13-acetate (Sigma-Aldrich) for 24 h. This was followed by an overnight resting period in fresh RPMI medium. Differentiation was confirmed by observing cellular adhesion and spreading.

Human PDLFs were provided as a kind gift from Dr. Douglas Hamilton's lab, Schulich's School of Medicine & Dentistry, London, ON, Canada. Cells were isolated from clinical samples of healthy periodontium. PDLFs were cultured in complete Dulbecco's Modified Eagle's Medium (DMEM) (Sigma-Aldrich), supplemented with 10% heat-inactivated fetal bovine serum and 1% antibiotic/antimycotic. The cells were maintained in a 37°C incubator with 5% CO_2_ in a humidified atmosphere. Experiments were conducted using cells from the third to fifth passage.

### Cell viability assay

To determine the optimal concentration of wine, both DRW and W (Vinoh, Brazil) were opened under sterile conditions within a biosafety cabinet and immediately filtered using a vacuum filtration membrane. The wines were then diluted in complete DMEM to concentrations of 15, 1, and 0.1%.

PDLFs monocultures were exposed to the test media for 24 h. Cells cultured in complete DMEM alone served as the negative control. The media was removed, and triplicate samples of cells were incubated with 200 µL of calcein-AM (Invitrogen/Molecular Probes, USA) for 20 min. Fluorescence was observed at 10× magnification using a fluorescent inverted microscope (Vert.A1; Carl Zeiss, Germany). Images were acquired and analyzed using ImageJ software (National Institutes of Health, USA). Cell viability was calculated as a percentage relative to the negative control, which was considered to be 100% viable (20).

Based on the PDLFs viability results, the most suitable wine concentration was tested in MQ monocultures following the same viability assay protocol. The cytokine and chemokine analysis were performed only after confirming the viability of MQ at the selected concentration.

### Assessment of cytokine and chemokine profile

After the determination of optimal concentration, cell culture supernatants of MQ and PDLFs co-culture treated with DMEM (C), 0.1% concentration of DRW (0.1% DRW), or W (0.1% W) were collected at 24 h. The samples were then centrifuged at 10,000 *g* and 4°C for 5 min to remove residual cells, aliquoted, and stored at -80°C until analysis. A multiplex immunoassay was conducted to simultaneously analyze the inflammatory cytokines interleukin (IL)-1β, tumor necrosis factor (TNF)-α, IL-8 (a chemokine), IL-10, IL-6, and vascular growth factor A (VEGF-A) using the HCYTOMAG-60K Milliplex MAP Human cytokine magnetic bead panel (Millipore, USA). The assessment was performed in duplicate. Data are reported in picograms per milliliter (pg/mL).

### Animal experiment

Once approved by the Ethics Committee (Araçatuba School of Dentistry, São Paulo State University, No. 0221-2022), twenty-four male rats (*Rattus norvegicus*, *Wistar*), aged 3 months and weighing 300-350 g each, were housed in a temperature-controlled environment (22°C±1°C, 70% humidity) with a 12-h light-dark cycle. The animals were fed a solid diet and water *ad libitum* throughout the experimental period. Solid diet was not provided during the 12 h leading up to the intervention. The sample size was based on previous studies recommending at least seven animals per group (12-14). To account for potential complications, one extra animal was added per group, totaling 24 animals (n=8 per group).

The animals were randomly assigned to three groups as follows: Control (C), rats receiving 4.28 mL/kg body weight of potable water to mimic the same procedure endured by the other animals; DRW, rats receiving 4.28 mL/kg body weight of DRW containing 0.3% ethanol by volume; and W, rats receiving 4.28 mL/kg body weight of W containing 12.5% ethanol by volume (12-14). This amount of the test solutions is considered moderate consumption for a male individual (300 mL per day) (21). The solutions were administered daily through oral gavage for 45 days supplementing the conventional diet. This regimen began 15 days before the induction of AP and continued for an additional 30 days (12,13). All animal experiment protocols were performed in accordance with the ARRIVE guidelines for reporting *in vivo* experiments.

### Induction of AP

On the 15th day, all animals were anesthetized with 80 mg/kg ketamine (Avenco Inc., USA) and 4 mg/kg xylazine (Mobay Corp., USA) and received AP induction. The first and second maxillary and mandibular molars on the right side had the pulp exposed using a 0.5 mm diameter carbon steel bur (Long Neck Ln Bur, Maillefer, Dentsply, Switzerland), the coronal openings were standardized and the teeth remained open until the end of the experiment (6,9,13). After 30 days from induction, the periapical lesion development was confirmed through digital radiography.

### Blood profile and assessment of inflammatory/bone markers

Thirty days after inducing AP, the animals were anesthetized following the previously described protocol. A cardiac puncture was performed to collect 5 mL of blood. The blood samples were then placed in EDTA and homogenized for further processing. An aliquot was separated for the blood profile analysis. Afterward, the blood was centrifuged (1,710 *g*, 4°C, 5 min) to separate the plasma. The plasma samples were transferred to Eppendorf tubes for enzyme-linked immunosorbent assay (ELISA) multiplex analysis.

The blood count parameter of leukocytes and absolute differential count of neutrophils, lymphocytes, monocytes, and eosinophils (7) were determined using an automated hematology analyzer (Mindray - BC 2800 Vet; Shenzhen Mindray Animal Medical Technology Co., LTD., China).

For the multiplex analysis, the MLLIPLEX^®^ Rat Cytokine/Chemokine Magnetic Bead Panel - Immunology Multiplex Assay (RECYTMAG-65K, Merck Life Science, LLC, Germany) was used for the quantification of IL-10, IL-1β, IL-17A, and TNF-α, and the MILLIPLEX^®^ Rat Myokine Magnetic Bead Panel (RMYOMAG-88K, Merck Life Science, LLC) was used for the detection of osteocrin and secreted protein acidic and rich in cysteine (SPARC). All analyses were performed in duplicate. Data are reported in pg/mL (22).

### Radiographic, qRT-PCR, and histometric analyses of AP

The animals were euthanized using an anesthetic solution overdose to collect the right-side maxillae and jaws for radiographic, quantitative reverse transcription polymerase chain reaction (qRT-PCR), and histometric analysis of AP.

The maxillae (n=4) were processed for digital radiography using an X-ray equipment Spectro 70X (Dabi Atlante, Brazil), with a 70 kVp nominal voltage and 7.0 mA tube current, and a digital sensor (Acuity direct X-ray photon detection model 005-000124) to confirm AP development. Radiographic images were acquired with an exposure time of 0.8 s and a total focal distance of 20 cm. The lesion size was measured using ImageJ Software (National Institutes of Health). The periapical lesion borders and size around the mesial roots of the first molars were marked, and the area was calculated in square millimeters and compared between groups (23).

For the qRT-PCR analysis, a bone block was removed from the AP site of the maxillae (n=4) using a 2-mm trephine bur (Trephine Bur 2.0 mm, Harte Surgical Instruments, Brazil). The collected bone blocks were immediately pulverized in liquid nitrogen and homogenized in TRIzol^®^ using a mortar and pestle to ensure complete tissue dissociation for RNA extraction. Total RNA was then extracted (PureLink™ RNA Mini Kit, Thermo Fisher Scientific, USA), DNase I-treated, and purity confirmed by 260/280 (1.8-2.0) and 260/230 (2.0-2.2) ratios. RNA concentration was measured by NanoDrop (Thermo Fisher Scientific), and 2 µg were reverse‐transcribed to cDNA (High-Capacity RNA-to-cDNA™ Kit, Applied Biosystems, USA). The gene expression analysis of IL-1β, IL-10, and TNF-α was performed in duplicate using the StepOne Plus™ Real-Time PCR System and TaqMan Gene Expression Assays (Applied Biosystems and Thermo Fisher Scientific). Target gene expression was normalized to Actb and the relative abundance of transcripts determined using the 2^-ΔΔCt^ method (24), with data reported as relative expression.

For the histometric analysis, the jaws (n=8) were demineralized in a 10% EDTA solution, embedded in paraffin and sectioned at 5 µm thick following a general histology protocol. Hematoxylin-eosin staining was used to assess differences in periapical bone loss among the different groups. The periapical area associated with the distal root of the first molar, defined by tracing the lesion boundary, was measured and reported in square millimeters using Leica Microsystems Software (Leica, Germany) (25).

### Statistical analysis

Statistical analyses were performed using GraphPad Prism 8.0.1 (GraphPad Software, USA). For parametric data, one-way analysis of variance followed by Tukey's *post hoc* test was applied. For non-parametric data, the Kruskal-Wallis test was used, followed by Dunn's *post hoc* test. Comparisons between two groups were conducted using Student's *t*-test. Results were considered statistically significant when P<0.05.

## Results

### Cell viability assay

**Figure 1:**
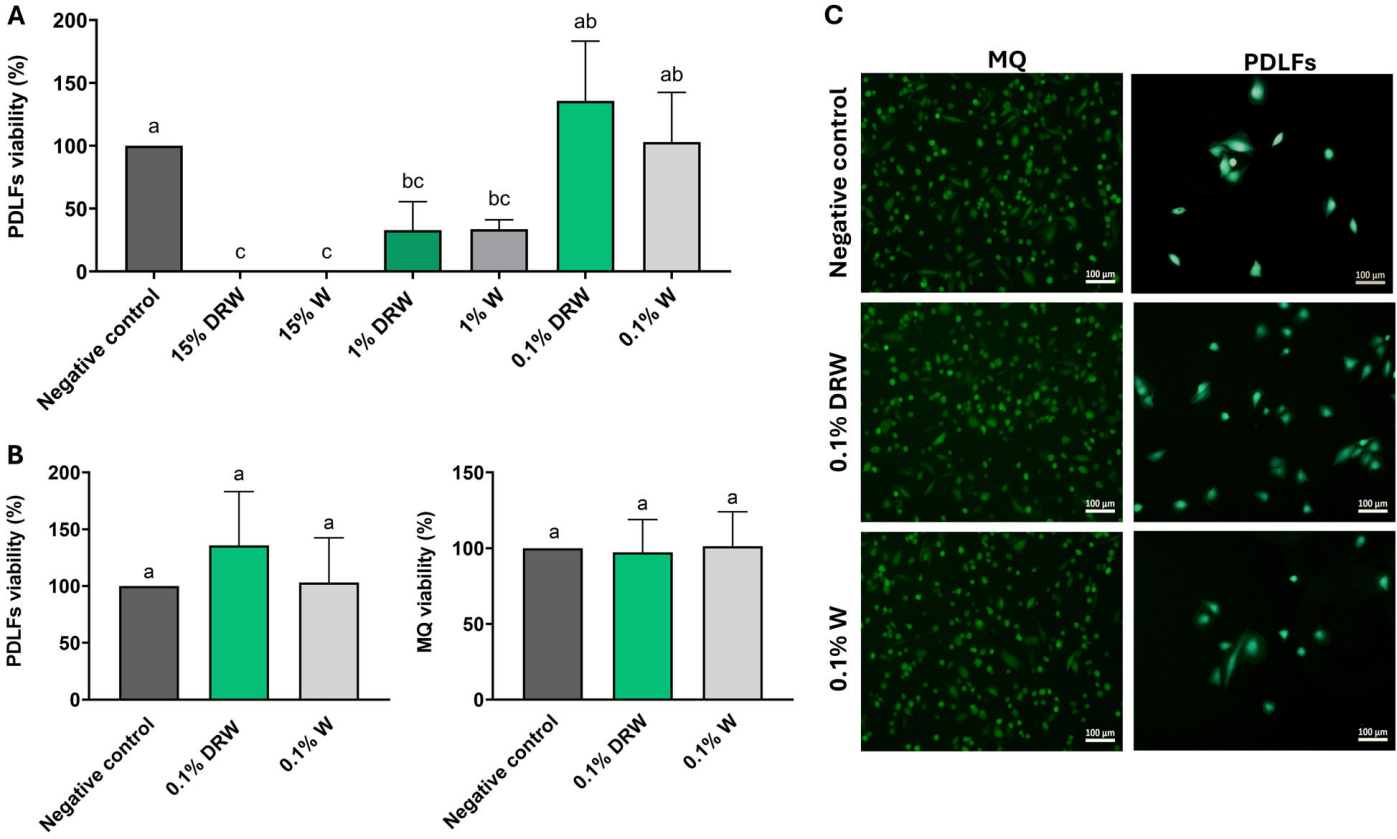
**A,** Cell viability of periodontal ligament fibroblasts (PDLFs) under different concentrations of dealcoholized red wine (DRW) and red wine (W) relative to the negative control. **B,** Cell viability of macrophages (MQ) and PDLFs monocultures under 0.1% of dealcoholized red wine (0.1% DRW) or red wine (0.1% W) relative to the negative control. Data are reported as means and SD. One-way ANOVA with Tukey *post hoc* test was applied. Different lowercase letters indicate significant difference among the groups (P<0.05). **C,** Calcein AM-stained images (10× magnification, scale bar=100 μm) at 24 h of MQ and PdLFs monocultures under 0.1% DRW or 0.1% W.

Figure 1 shows the cell viability results for PDLFs after 24 h of exposure. Higher concentrations of DRW and W (15 and 1%) significantly reduced cell viability compared to the negative control (P<0.05). A dose-dependent improvement in viability was observed as concentrations decreased, with the 0.1% concentration maintaining viability at levels comparable to the control group. Based on these results, 0.1% was selected for subsequent experiments. Both MQ and PDLFs exposed to 0.1% DRW and 0.1% W remained viable, with no significant differences relative to the control group (P>0.05).

### Cytokine and chemokine profile

**Figure 2:**
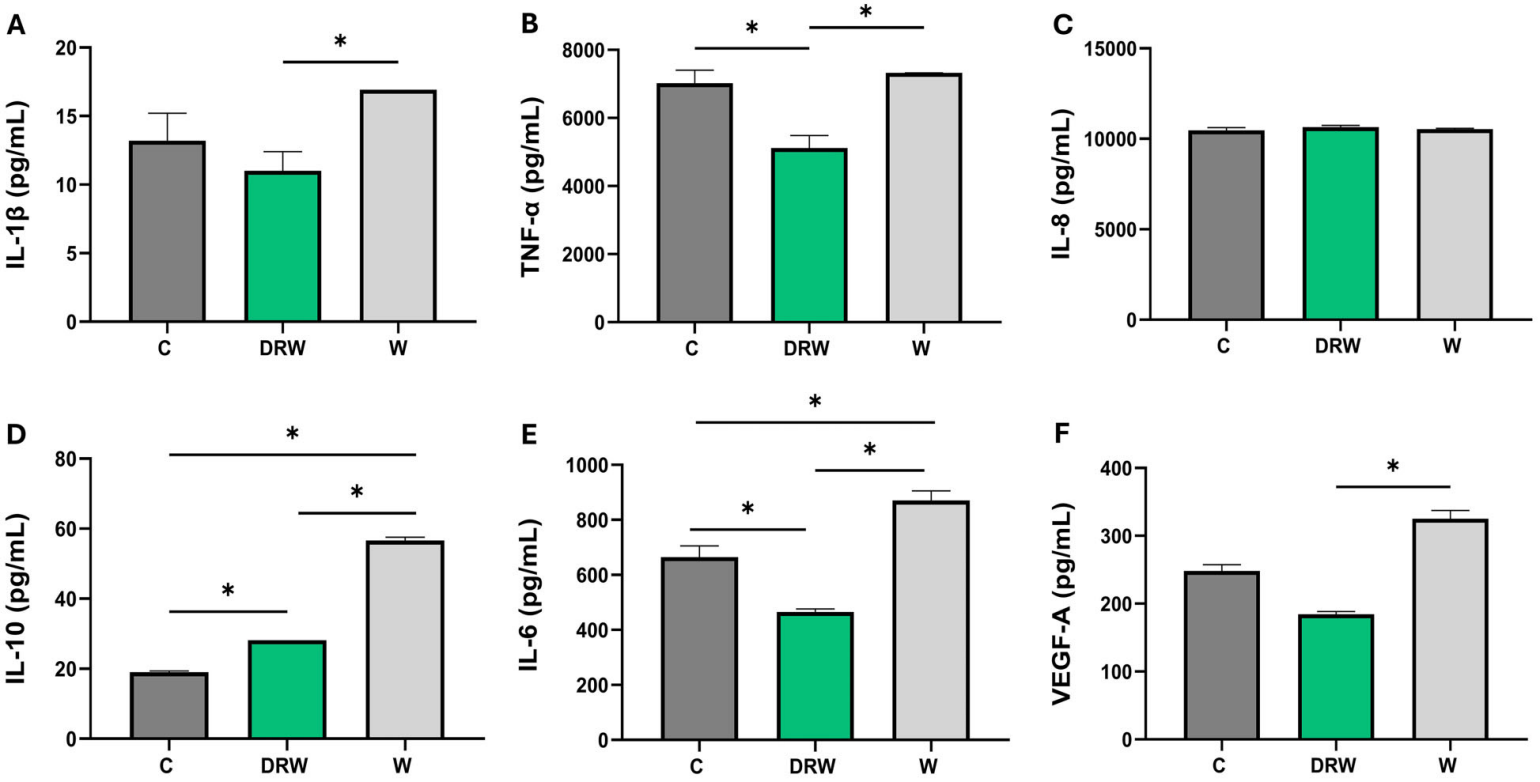
The bar graphs show the mean and standard deviation for the assessment of **A,** IL-1β; **B,** TNF-α; **C,** IL-8; **D,** IL-10; **E,** IL-6; and **F,** VEGF-A for the co-culture of macrophages (MQ) and periodontal ligament fibroblast (PDLFs). Data are reported as means and SD. *P<0.05; one-way ANOVA with Tukey *post hoc* test was applied.

Figure 2 presents the cytokine and chemokine profile from the co-culture of MQ and PDLFs. DRW exhibited significantly reduced IL-6 and TNF-α levels compared to C and W, higher IL-10 level compared to C, and lower IL-1β level only compared to W (P<0.05).

### Blood profile analysis

The results of the blood profile analysis are presented in Table 1. No significant difference was observed in leukocyte count among the groups. However, the absolute differential count revealed a significantly lower monocyte count for DRW compared to C (P<0.05), but similar to W (P>0.05). The counts of neutrophils, lymphocytes, and eosinophils showed no significant difference among the groups (P>0.05).

**Table 1:** Blood profile of the study groups.

Blood parameters	Groups		
	C	DRW	W
Leukocytes (×10^3^/µL)	9.131±1.871^a^	8.925±1.480^a^	8.593±1.132^a^
Neutrophils (cells/µL)	1.868±0.748^a^	1.884±0.683^a^	1.814±0.432^a^
Lymphocytes (cells/µL)	6.711±1.278^a^	6.770±0.988^a^	6.307±0.951^a^
Monocytes (cells/µL)	404.4±160.5^b^	209.8±157.6^a^	356.3±135.2^a,b^
Eosinophils (cells/µL)	109.9±75.69^a^	146.3±54.77^a^	103.2±119.7^a^

Data are reported as means±SD. Different superscript lower-case letters in rows indicate a significant difference among the groups (P<0.05, one-way ANOVA followed by Tukey's test for multiple comparisons). C: control; DRW: dealcoholized red wine; W: red wine.

### Assessment of inflammatory/bone markers

**Figure 3:**
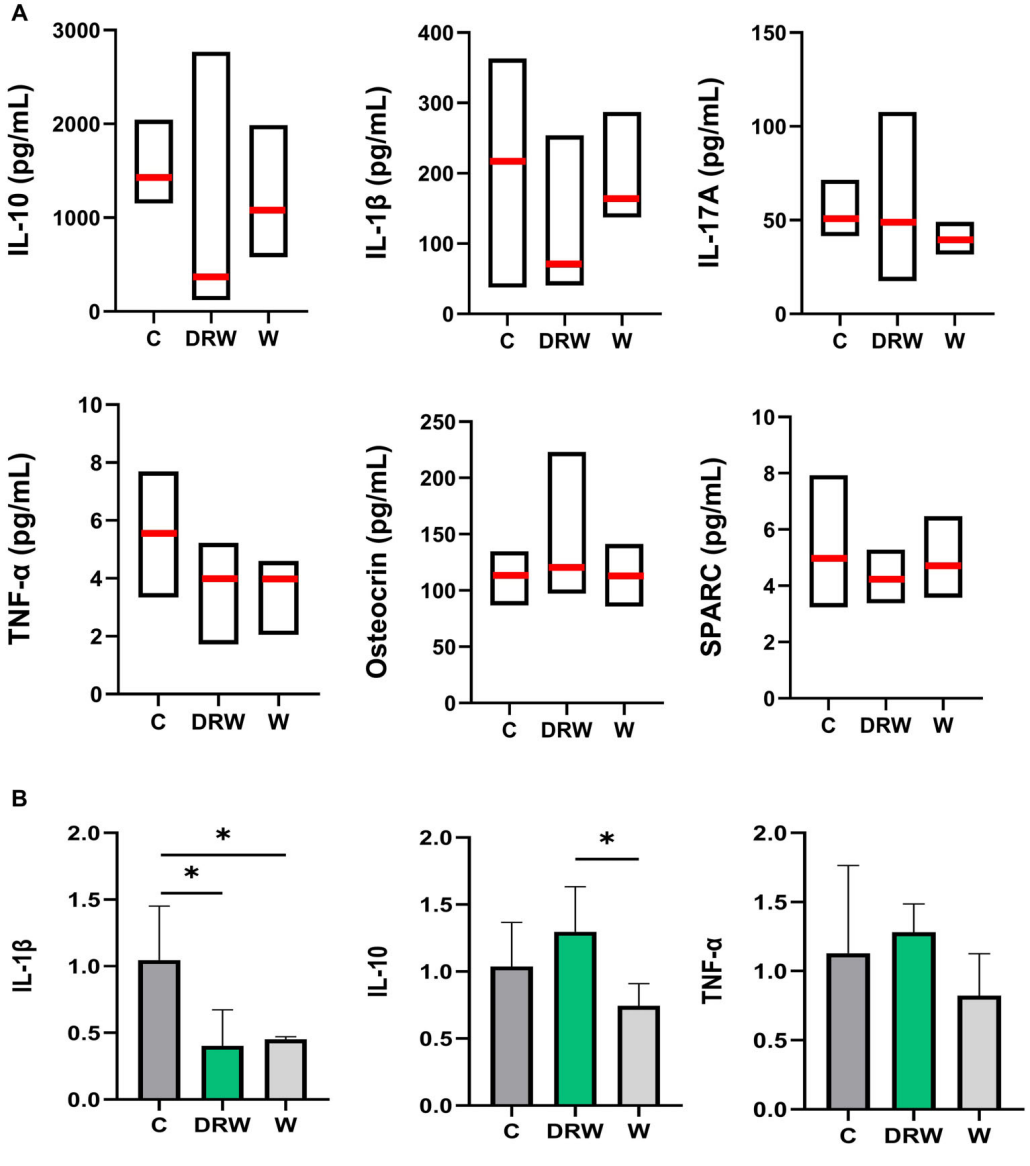
A, Median (red line), minimum, and maximum values for interleukin (IL)-10, IL-1β, IL-17A, tumor necrosis factor (TNF)-α, osteocrin, and secreted protein acidic and rich in cysteine (SPARC) in plasma samples for each group. Kruskal-Wallis test followed by Dunn's *post hoc* test was applied (P>0.05). **B**, Mean and standard deviation of relative gene expression (2^-ΔΔCt^) from qRT-PCR analysis of apical periodontitis samples for IL-1β, IL-10, and TNF-α. *P<0.05; one-way ANOVA with Tukey *post hoc* test was applied.

The systemic analysis of plasma cytokines showed comparable levels of IL-10, IL-1β, IL-17A, TNF-α, osteocrin, and SPARC among the groups (P>0.05) (Figure 3A), indicating a stable systemic cytokine profile under the experimental conditions.

### Radiographic analysis of AP

**Figure 4:**
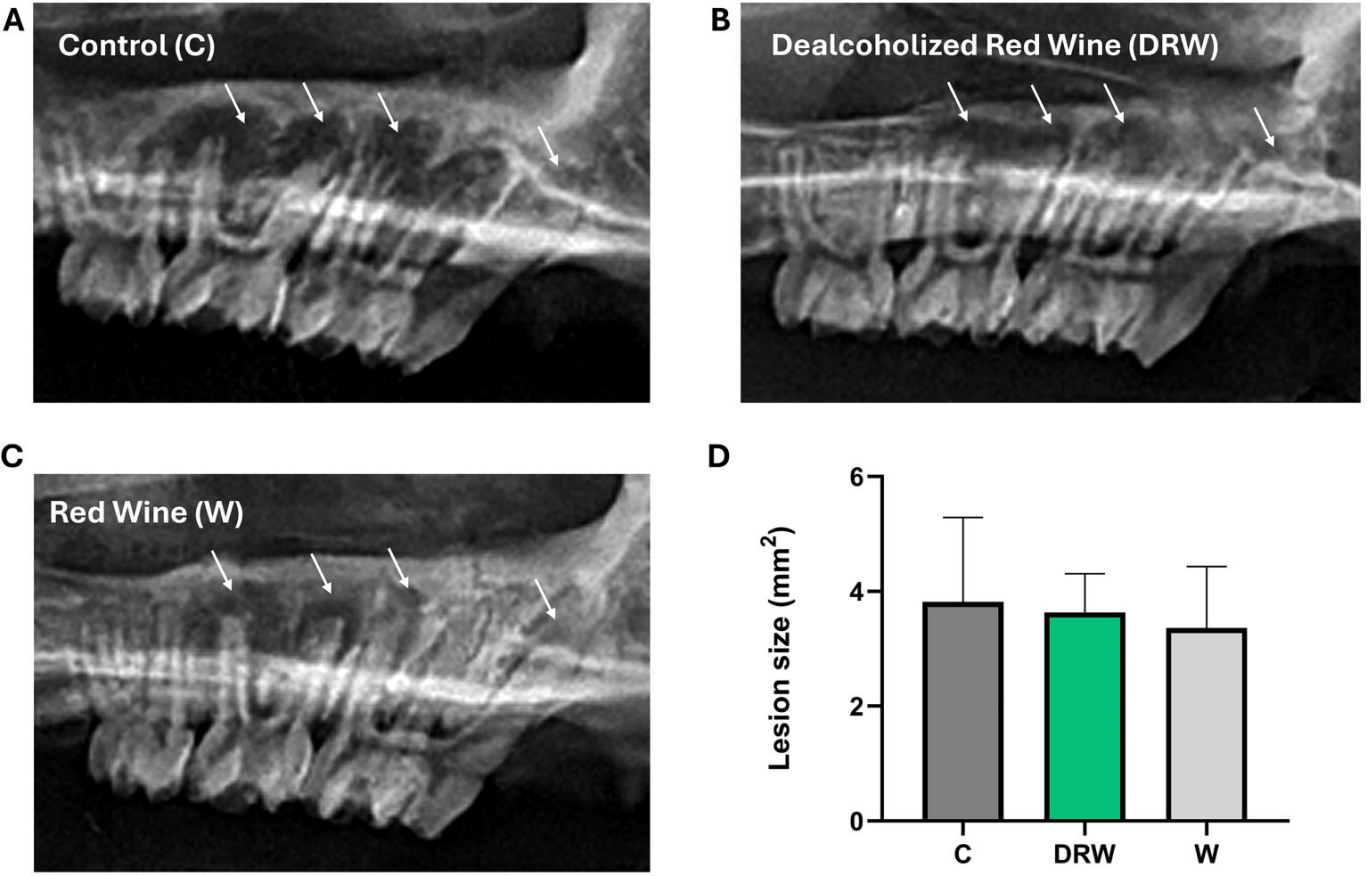
Representative radiographic images from the specimens in the **A,** Control; **B,** dealcoholized red wine (DRW); and **C,** red wine (W) groups. The white arrows indicate the periapical lesions in the first and second maxillary molars. **D,** The bar graph for lesion size shows the mean and standard deviation in mm^2^ of each group. There were no significant differences among groups (P>0.05). One-way ANOVA with Tukey *post hoc* test was applied.

Radiographic imaging of the maxillae confirmed the development of AP in all groups (Figure 4). The periapical lesion areas were measured to assess lesion size, and no statistically significant differences were detected among the groups (P>0.05), indicating that the tested supplementations did not alter radiographic lesion size under the present experimental conditions.

### qRT-PCR analysis of AP

The graphs for the qRT-PCR analysis are shown in Figure 3B. The AP sites in the jaws of DRW and W showed significantly lower IL-1β expression compared to C (P<0.05). Additionally, IL-10 expression was higher for DRW only compared to W (P<0.05). No significant difference was observed in TNF-α expression among the groups (P>0.05).

### Histometric analysis of AP

Table 2 presents the histometric analysis of AP for all groups. In all groups, histological sections showed evident periapical lesions characterized by bone resorption surrounding the distal root of the first molar. The lesion boundaries were clearly delimited and measured to calculate the periapical area. The periapical bone loss area was similar among groups (P>0.05).

**Table 2:** Area of apical periodontitis in each study group.

Group	Histometric analysis (mm^2^)
C	2283.976±310.672
DRW	2099.349±671.320
W	2094.260±563.611

Data are reported as means±SD. P>0.05, one-way ANOVA followed by Tukey's test for multiple comparisons. C: control; DRW: dealcoholized red wine; W: red wine.

## Discussion

The findings of this study support the hypothesis that DRW and W differentially modulate the inflammatory profile by influencing key cytokines and immune cell responses. In co-cultures of MQ and PDLFs, DRW promoted a more anti-inflammatory profile than W, characterized by decreased TNF-α and IL-6, with increased IL-10 levels. *In vivo*, DRW intake led to a lower blood monocyte count and reduced IL-1β expression at the AP site, contributing to an anti-inflammatory environment.

After determining the optimal concentration, both DRW and W at 0.1% showed no cytotoxic effects on MQ and PDLFs. These cells play key roles in the development and progression of AP (26). MQ are innate immune cells that participate in inflammation by balancing the production of pro- and anti-inflammatory cytokines, such as IL-6, TNF-α, and IL-10, which in turn influence bone resorption and repair (27,28). PDLFs, resident cells of the periodontal ligament, contribute to tissue homeostasis by maintaining the extracellular matrix and regulating cytokine production through crosstalk with MQ, modulating the immune response (29,30). In addition, they influence bone remodeling by stimulating osteoclast precursors and regulating the expression of receptor activator of nuclear factor-κB ligand and osteoprotegerin, key mediators of osteoclastogenesis and bone turnover (31).

The use of a direct co-culture system allowed to model cell-to-cell interactions occurring in the periapical environment. In this setting, DRW promoted an anti-inflammatory response by suppressing TNF-α and IL-6, while increasing IL-10 levels. Interestingly, IL-1β, TNF-α, IL-6, IL-10, and VEGF-A levels were increased in W compared to DRW.

The anti-inflammatory effects of DRW are likely mediated by polyphenols such as resveratrol, quercetin, and other flavonoids. These compounds modulate signaling pathways in MQ, including nuclear factor-kappaB (NF-κB) and p38 mitogen-activated protein kinase (p38 MAPK), which regulate cytokines such as IL-1β, TNF-α, and IL-6 (32). In lipopolysaccharide (LPS)-stimulated PDLFs, resveratrol suppresses TNF-α, IL-6, and IL-1β in a concentration- and time-dependent manner (33). Inhibition of TLR4-NF-κB/MAPK/IRF3 signaling by resveratrol has also been shown to reduce TNF-α and IL-6 while increasing IL-10 (34). Moreover, polyphenols modulate MAPK and oxidative stress pathways, contributing to the attenuation of pro-inflammatory cascades (35). Since VEGF is often induced by these cytokines (36), its upregulation may enhance vascular permeability and leukocyte infiltration. Differences in responses between DRW and W may be partly due to ethanol in W, which can promote pro-inflammatory signaling (15-17).

As a key mediator of periodontal inflammation, IL-1β promotes immune cell recruitment and bone resorption in AP (3). Therefore, its downregulation observed *in vivo* aligns with reduced circulating monocytes, that migrate to site of injury and differentiate into MQ, regulating both the initiation and maintenance of chronic inflammation (37). Additionally, the higher expression of IL-10 in DRW-treated animals compared to W reinforces its potential regulatory role. These results agree with previous studies where a combination of resveratrol with quercetin or DRW supplementation led to higher IL-10 and reduced IL-1β immunolabeling in developing and established AP models (12,14).

Despite the observed molecular changes, ELISA revealed no significant differences among groups in blood levels of IL-10, IL-1β, IL-17A, TNF-α, osteocrin, and SPARC. As previously reported, the inflammatory effects in blood cytokine levels tend to be more evident in animals with multiple foci than those without AP or with a single lesion (6-8), potentially masking anti-inflammatory systemic effects of the tested supplementations. Furthermore, plasma cytokine levels likely reflect a cumulative, time-dependent systemic response, indicating that systemic changes in blood samples of rats with AP can be subtle, transient, and vary with disease progression (38).

Moreover, DRW supplementation did not result in statistical differences in lesion size/periapical bone loss among groups assessed through radiographic and histometric analyses. This may reflect the complexity of bone remodeling, which depends not only on local cytokine levels but also on systemic factors, mechanical influences, and lesion stage, and is tightly regulated by a network of mediators such as IL-1β, TNF-α, IL-6, IL-17, IL-10, and the RANKL/OPG axis (39,40). In the present model, the AP stage may have been too early to allow detectable changes in lesion size or bone loss, since the inflammatory process was still under development. Importantly, evaluating more advanced stages of AP could be key to uncovering the modulatory effects of DRW polyphenols on bone resorption, particularly since differences in lesion size have been documented after 60 days of disease progression (14). These findings suggest that while DRW polyphenols can influence the inflammatory environment, additional or more sustained interventions may be required to translate these cellular effects into reductions in bone pathology.

Some limitations of this study should be noted. *In vitro*, assessing MQ polarization markers and including bacterial stimuli could clarify cellular pathways and inflammatory responses. *In vivo*, evaluating mediators such as the RANKL/OPG ratio may explain why IL-1β suppression and IL-10 upregulation did not reduce bone loss. Additionally, early peaks in cytokine expression may have been diminished at the 45-day assessment (40). Finally, isolating specific polyphenols could identify the main active components, guiding targeted therapies.

Considering the recent nature of research on the anti-inflammatory effects of DRW in AP, and the complex interplay of local and systemic immune responses involved in the disease, these findings should be interpreted with caution and are not directly translatable to clinical settings. Nonetheless, this study offers valuable insights into the immunomodulatory potential of DRW in both *in vitro* and *in vivo* models, highlighting the need for further investigations to better elucidate its mechanisms of action.

In conclusion, DRW and W exerted distinct effects in both co-culture and *in vivo* AP models. DRW promoted a pronounced anti-inflammatory profile by reducing TNF-α and IL-6 while increasing IL-10 in co-cultures, and by lowering blood monocyte counts and decreasing IL-1β expression *in vivo*. However, these anti-inflammatory effects were not sufficient to alter bone disease progression within the experimental model evaluated.

## Data Availability

All data generated or analyzed during this study are included in this published article.
